# Early-Onset Cancers in Adults: A Review of Epidemiology, Supportive Care Needs and Future Research Priorities

**DOI:** 10.3390/cancers14164021

**Published:** 2022-08-20

**Authors:** Ashleigh C. Hamilton, David W. Donnelly, Deirdre Fitzpatrick, Helen G. Coleman

**Affiliations:** 1 Centre for Public Health, Queen’s University Belfast, Belfast BT12 6BA, UK; 2 Northern Ireland Cancer Registry, Queen’s University Belfast, Belfast BT12 6DP, UK; 3 Patrick G. Johnston Centre for Cancer Research, Queen’s University Belfast, Belfast BT9 7AE, UK

**Keywords:** early-onset cancer, young onset cancer, epidemiology, supportive care needs

## Abstract

**Simple Summary:**

Early-onset cancers, defined as cancers in adults aged 18 to 49 years, are increasing in a number of cancer sites in developed countries. Cancers commonly seen in older people are now being diagnosed in younger adults, for example bowel, breast, stomach and pancreatic cancers. In this review, we report statistics about early-onset cancers using exemplar data from a UK region and discuss issues unique to this age group. Topics covered include the long-term consequences of cancer treatment, how cancer treatment affects fertility and the use of social media by patients, healthcare professionals and researchers. We also outline important future research priorities for early-onset cancers.

**Abstract:**

Rising incidence of specific types of early-age onset cancers in adults aged 18–49 years has been reported in high-income countries. In this review, we summarise the epidemiology of early-onset cancers using exemplar data from a high-income UK region, discuss supportive care needs for young patients and outline future research directions. The incidence rate of early-onset cancers increased by 20.5% from 1993 to 2019 in Northern Ireland. Differences in types of cancer were observed between sexes and across age groups of 18–29, 30–39 and 40–49 years. One and five-year net survival was mostly better in 18–29-year-olds for all cancers combined compared to older age groups for both sexes, but there were variations in specific cancer types. Poorer survival was observed for patients with brain/central nervous system, connective and soft tissue or lung cancers. Patients with early-onset cancers face unique supportive care needs and require holistic care. The impact of cancer treatment on fertility and fertility preservation treatments is an important consideration. Social media can be used for patient support, information, fundraising, advocacy work and recruitment to research studies. We also outline suggested future research priorities for early-onset cancers, spanning prevention, diagnosis, treatment and supportive care needs.

## 1. Introduction

According to Globocan statistics, there were an estimated 19.3 million new cancer cases and approximately 10 million cancer deaths worldwide in 2020 [1]. Analysis of trends in the UK regarding the risk of cancer have quantified that one in two people in the UK born after 1960 will develop cancer in their lifetime [2]. A proportion of this increased risk compared to previous generations is due to increasing life expectancy, with over one-third of new cases of cancer in the UK from 2016 to 2018 occurring in people aged 75 years and over [3]. However, the largest proportional increase in incidence rates of all cancers combined since the early 1990s in the UK has been observed in people aged 25 to 49 years. European age-standardised (EAS) incidence rates have increased from 132.7 per 100,000 in 1993–1995 to 162.4 per 100,000 in 2016–2018, representing a 22% increase in 25 to 49 year olds [3]. By comparison, the proportional increase in incidence rates in 50 to 74 yearolds is 13%, and 9% in those aged 75 and over. Globally, cancer was the fourth most common cause of death in 15 to 39yearolds in 2019, with only self-harm/violence, transport injuries and cardiovascular diseases causing a higher number of deaths [4]. The most common cancers vary by age group and sex. For example, it is well recognised that testicular cancer and cervical cancer tend to occur in adolescents and younger adults, whereas prostate cancer commonly occurs in men aged over 50 years. However, recent evidence shows that cancers historically perceived to be more common in older age groups are now being diagnosed in younger adults. Increases in the incidence of colorectal cancer [5], breast cancer [6], oesophageal adenocarcinoma [7], gastric cancer [8], uterine cancer [9], kidney cancer [9] and pancreatic cancer [10] in younger age groups have been reported in high-income countries.

Some of these trends have been reported on by mainstream media outlets such as the BBC and the *Guardian* [11,12,13]. Recent high-profile deaths of celebrities with cancer, such as British singer Sarah Harding who died from breast cancer at 39, British singer Tom Parker who died from glioblastoma at 33 and American actor Chadwick Boseman who died of colorectal cancer at 43 have also been reported in the media. Dame Deborah James, also known as bowelbabe on social media, publicly chronicled her cancer journey in books, podcasts, a national newspaper column and on social media following a diagnosis of colorectal cancer at 35 in 2016. At the time of her death in June 2022, her awareness campaign had raised several million pounds for research and was covered as a top news story in the UK. These public experiences have served to raise awareness of cancer in younger people, symptoms of cancer and the experiences of younger adults living with cancer, including end-of-life care.

Urgent research is required to understand the likely multifactorial reasons for the changing incidence trends and patterns of cancers within adults under 50. A study investigating the prevalence of germline mutations in early-onset cancer (cancers that do not typically occur in younger adults aged 18 to 39 years—the commonest ones being colorectal, breast, kidney, pancreatic and ovarian cancer) found that 21% of cases harboured a germline mutation [14]. Lifestyle factors, for example obesity and alcohol intake, are associated with several cancers, and as rates of obesity are increasing in many countries [15], these modifiable risk factors may be playing a role.

The observed incidence trends also serve as a warning about the future disease burden for healthcare systems and indicate areas for future planning in terms of resources and policy. The COVID-19 pandemic has resulted in unparalleled pressure on healthcare systems internationally, with many currently stretched beyond capacity and facing years of recovery and a backlog of waiting lists. It is imperative, therefore, that action is taken now to address the observed rise in early-onset cancers in the areas of prevention, early detection and treatment.

In this narrative review article, we summarise the epidemiology of early-onset cancers diagnosed since 2010 in adults. We present exemplar data from Northern Ireland, a high-income region in which the UK National Health Service free healthcare at the point-of-access applies, discuss supportive care needs in this age group and summarise future research directions.

## 2. Epidemiology

In this section we outline the descriptive epidemiology of incidence and survival of early-onset cancers in Northern Ireland, using data from the Northern Ireland Cancer Registry (NICR).

### 2.1. Methods

Data on all cancer cases (excluding non-melanoma skin cancer(NMSC)) diagnosed in NI between 1993 and 2019 were extracted from the NICR; a population-based registry with 100% coverage of the Northern Ireland population of c.1.9 million people [16]. The NICR is the officially recognised provider of cancer statistics for Northern Ireland, and the high quality of the NICR data, including their validity and completeness have been previously reported [17]. The three main sources for cancer registration are the Patient Administration System used by all the Hospital Trusts, pathology reports from Trusts and death notifications which are supplied by the General Register Office. NICR has ethical approval from the Office of Research Ethics Northern Ireland for collection of the data used in this study.

Cancer data were coded using the International Classification of Diseases (ICD-10) [18], with codes C00–C97 (excluding C44) used to identify relevant cases, and specific cancer types were classified using the codes specified in Supplementary Table S1.

### 2.2. Statistical Methods

Extracted data were initially used to produce counts of the number of cases each year in the 18 to 49 age group, along with the age groups: 18 to 29, 30 to 39 and 40 to 49 years. Due to the small number of cases for specific cancer types, 10 years of data (2010–2019) were pooled when investigating the number of cases by cancer type. Results are presented as an average number of cases per year, being the total number of cases divided by the number of years rounded to the nearest integer.

Due to the relationship between cancer and age, when investigating variations in incidence over time and by cancer type, results were directly age-standardised to provide a rate per 100,000 persons. Northern Ireland population data used in this calculation were provided by the Northern Ireland Statistics and Research Agency [19], while the 2013 European standard population was used to provide the weights for each five-year age group (including the first age group which was 18–19 years).

The net survival method was used to estimate cancer survival, specifically the Pohar-Perme method [20] with the stns Stata command [21], as this approach provides survival of patients if cancer was the only possible cause of death. Follow-up of patients was available until the end of 2019, and 15 years’ worth of data were used in order to provide a sufficient number of cases to provide a robust survival estimate. Exclusions were made for death certificate only cases, while in the event that a patient had more than one type of the cancer being examined, the first event only was included. Background lifetables used in the calculation were the official lifetables for Northern Ireland, calculated on an annual basis by the Office for National Statistics [22].

Both age-standardised incidence rates and net survival estimates are accompanied by 95% confidence intervals (CI) which were used to make comparisons between groups.

### 2.3. Early-Onset Cancer Incidence over Time

**Figure 1:**
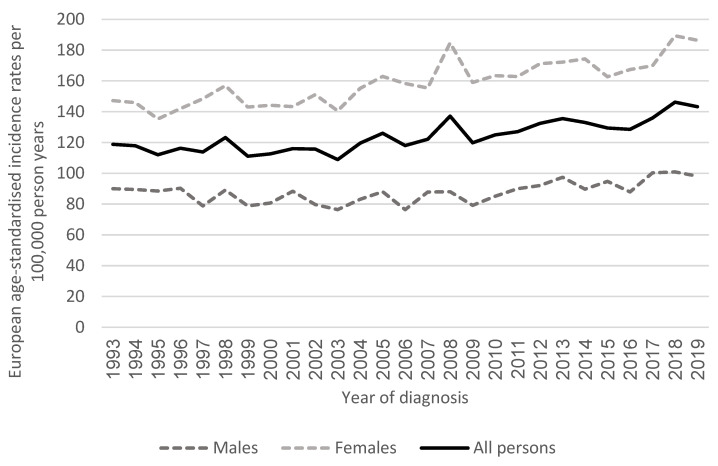
Age-standardised incidence rates of cancer (excluding non-melanoma skin cancer) by year of diagnosis in Northern Ireland in adults aged 18–49 years.

Within the population of Northern Ireland, we observed an increasing incidence of early-onset cancers, excluding NMSC, since 1993 in people aged 18 to 49 years, particularly from 2003 onwards (Figure 1), in keeping with reported trends seen in other high-income countries. In 2019, there were 1,076 cases of cancer in people aged 18 to 49 in Northern Ireland (excluding NMSC), with an EAS incidence rate of 143.2 per 100,000 person years (95%CI 134.7–151.8). This was a 20.5% increase compared to the EAS incidence rate of 118.8 per 100,000 person years in 1993 (95%CI 110.2–127.3).

### 2.4. Early-Onset Cancer Incidence by Sex

The EAS incidence rate in females was higher than in males for all years recorded (186.4 vs. 98.1 per 100,000 person years in 2019), largely driven by breast cancer diagnoses. From 1993 to 2019, there was a 27% increase in the EAS incidence rate of cancers (excluding NMSC) in females compared to a 9% increase in incidence in males for this time period. There were some differences in the types of cancer occurring in males and females aged 18 to 49 years, although lymphoma, melanoma, colorectal, lung and head and neck cancers were observed commonly in both sexes.

### 2.5. Males

**Figure 2:**
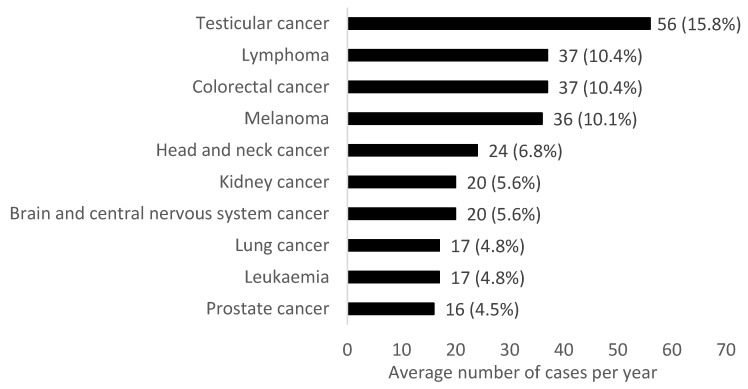
Average number of cases per year for the most common cancers (excluding non-melanoma skin cancer) in males aged 18–49 years in Northern Ireland from 2010–2019. Percentages refer to the proportion of the total average number of cancer cases per year.

The most common cancer in males aged 18 to 49 years from 2010–2019 was testicular cancer, followed by lymphoma and colorectal cancer (Figure 2). Differences in the common cancers were seen in age subgroups of 18–29, 30–39 and 40–49 years. The most common cancers in males aged 18–29 years were testicular, lymphoma and brain/central nervous system (CNS) cancers. The most common cancers in males aged 30–39 years were testicular cancer, melanoma and lymphoma. Finally, the most common cancers in males aged 40–49 years were colorectal, melanoma and head and neck cancer.

### 2.6. Females

**Figure 3:**
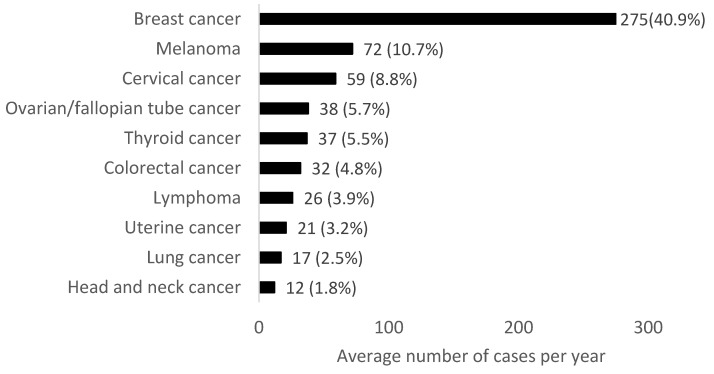
Average number of cases per year for the most common cancers (excluding non-melanoma skin cancer) in females aged 18–49 years in Northern Ireland from 2010–2019. Percentages refer to the proportion of the total average number of cancer cases per year.

In females aged 18 to 49 years in 2010–2019, the most common cancers were breast, melanoma and cervical cancer (Figure 3). In females aged 18–29 years the most common cancers were melanoma, cervical and thyroid cancers. In females aged 30–39 years, breast, cervical and melanoma were the most common cancers diagnosed. In females aged 40–49 years, the most common malignancies were breast, melanoma and colorectal cancers.

### 2.7. Implications

The pattern seen in Northern Ireland reflects a wider picture which is recognised in other high-income countries, with a changing spectrum of cancers across different age groups. According to a UK Cancer Statistics report in 2021, in adolescents the most common cancers are haematological, CNS and germ cell tumours along with other carcinomas and melanoma [23]. The malignancies observed in the 18–29-year age group in Northern Ireland are similar to those commonly found in adolescents, suggesting an overlap in epidemiology in these close age groups. In the older age group of 50 to 74 years in the UK, prostate, lung and colorectal cancers are the most commonly diagnosed in males, whereas in females, breast, lung and colorectal cancers are the most commonly diagnosed in this age group [3]. The malignancies frequently seen in the 40–49-year age group in Northern Ireland show a similar profile to older age groups, showing a change from the youngest age group studied. The 30–39-year age group in Northern Ireland displays an overlap of both the younger and older age groups, suggesting this is the decade where potential aetiologies change during the lifespan.

The incidence rate in females aged 18 to 49 years in Northern Ireland increased more than in males over the 26-year time period studied, with breast cancer accounting for an average of 41% of cancer cases per year in females. Cervical and ovarian/fallopian tube cancer are also the third and fourth most common cancers in the 18 to49-year age group. This suggests that consideration of risk factors should be sex-specific, and prevention and early detection measures may also need to be targeted to specific groups rather than an entire demographic.

### 2.8. Survival

In this section, we will discuss survival statistics for early-onset cancers in Northern Ireland. One-year and five-year net survival are for patients diagnosed from 2000–2014 and followed up to the end of 2019. Net survival is a survival estimate which has been adjusted to remove the impact of death from non-cancer-related causes.

### 2.9. Early-Onset Cancer Survival by Sex

Females had better survival outcomes than males for early-onset cancers (Supplementary Figure S1). One-year and five-year net survival for all cancers combined (excluding NMSC) were better in females than males in all three age groups studied (18–29, 30–39 and 40–49 years), although this was not statistically significant for 18–29-year-olds. Improved survival for females is likely due to the high proportion of breast cancer among females.

### 2.10. Males

Males in the youngest age group (18–29 years) had better one-year and five-year net survival for all cancers combined (excluding NMSC) compared to males aged 30–39 years, and in turn, males aged 30–39 years had better one-year and five-year net survival for all cancers combined (excluding NMSC) compared to those aged 40–49 years (Supplementary Figure S1).

The one-year and five-year net survival for males aged 18–29 years for all cancers (excluding NMSC) in Northern Ireland was 93.4% and 83.7%, respectively (Supplementary Table S2). Five-year net survival rates ranged from 41.0% for connective and soft tissue cancer to 95.5% for testicular cancer and 100% for thyroid cancer (based on the 10 most common cancer types in this age group).

The one-year and five-year net survival for males aged 30–39 years for all cancers (excluding NMSC) in Northern Ireland was 88.9% and 77.5%, respectively (Supplementary Table S3). Five-year net survival ranged from 49.5% for brain and CNS cancer to 99.5% for testicular cancer (based on the 10 most common cancer types in this age group).

The one-year and five-year net survival for males aged 40–49 years for all cancers (excluding NMSC) in Northern Ireland was 78.4% and 60.3%, respectively (Supplementary Table S4). Five-year net survival ranged from 17.2% for lung cancer to 93.9% for testicular cancer (based on the 10 most common cancer types in this age group).

**Figure 4:**
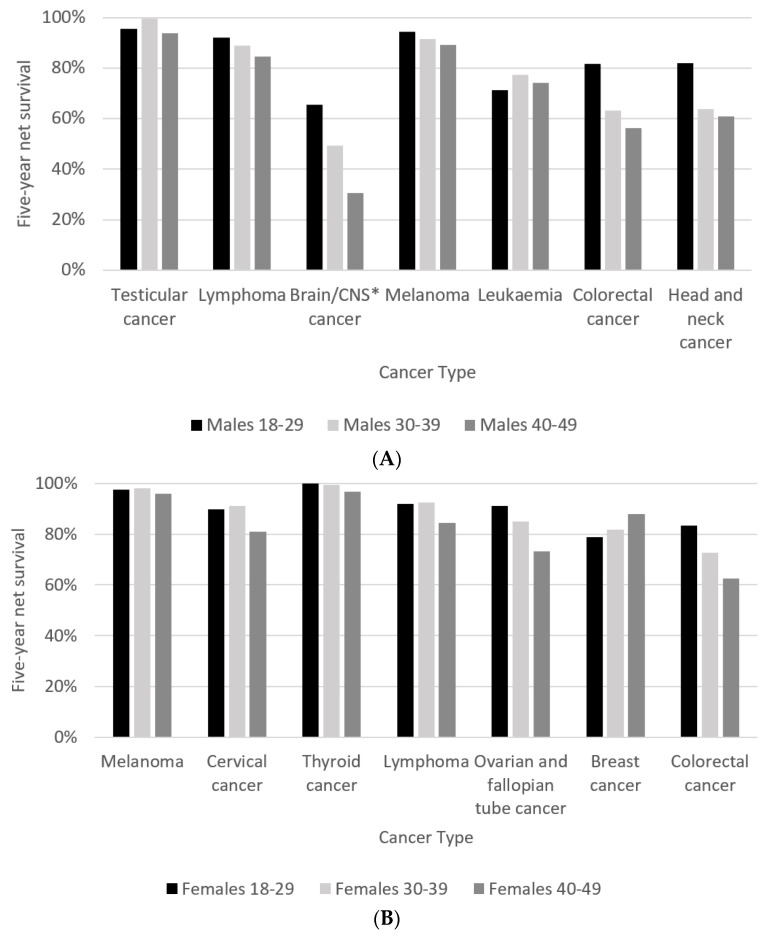
(**A**) Five-year net survival for males by age group diagnosed in 2000–2014, followed up to end of 2019. Cancer types displayed are the cancers common to all three age groups in males. * central nervous system; (**B**) five-year net survival for females by age group diagnosed in 2000–2014, followed up to end of 2019. Cancer types displayed are the cancers common to all three age groups in females.

For seven cancers that occurred commonly in all three age groups, the 30–39-year age group had the best five-year net survival for testicular cancer, while five-year net survival from brain and CNS cancer in this age group was better than in the 40–49-year age group (Figure 4A). Among those aged 40–49 years, five-year net survival was significantly poorer than in the 18–29-year age group for brain and CNS cancer, colorectal cancer and head and neck cancer. Other variations by age group existed but were not statistically significant in this analysis. One-year net survival for the seven cancers common to all three age groups is shown in Supplementary Figure S2.

### 2.11. Females

Females in the youngest age group (18–29 years) had better one-year and five-year net survival for all cancers combined (excluding NMSC) compared to females aged 30–39 years, and in turn, females aged 30–39 years had better one-year and five-year net survival for all cancers combined (excluding NMSC) compared to females aged 40–49 years (Supplementary Figure S1).

The one-year and five-year net survival for females aged 18–29 years for all cancers (excluding NMSC) in Northern Ireland was 94.9% and 87.1%, respectively (Supplementary Table S2). Five-year net survival rates ranged from 50.1% for connective and soft tissue cancer to 100% for thyroid cancer (based on the 10 most common cancer types in this age group).

The one-year and five-year net survival for females aged 30–39 years for all cancers (excluding NMSC) in Northern Ireland was 94.4% and 83.2%, respectively (Supplementary Table S3). Five-year net survival ranged from 37.6% for lung cancer to 99.4% for thyroid cancer (based on 10 ten most common cancer types in this age group).

The one-year and five-year net survival for females aged 40–49 years for all cancers (excluding NMSC) in Northern Ireland was 91.1% and 78.5%, respectively (Supplementary Table S4). Five-year net survival ranged from 18.1% for lung cancer to 96.7% for thyroid cancer (based on the 10 most common cancer types in this age group).

For seven cancers that occurred commonly in all three age groups, five-year net survival among 40–49-year-olds was significantly poorer than among 30–39-year-olds for cervical cancer, as well as ovarian and fallopian tube cancer (Figure 4B). It was also significantly poorer than for 18–29-year-olds for ovarian and colorectal cancers. However, five-year net survival for breast cancer among 40–49-year-olds was better than for 18–29 and 30–39-year-olds, with the latter difference being statistically significant. Further differences by age group were observed but were not statistically significant. Notably, breast cancer showed a better one-year net survival in 18–29-year-olds (Supplementary Figure S3), but 40–49-year-olds showed a better five-year net survival.

## 3. Discussion of Survival

Our results suggest that very young patients under 30 have better outcomes for a number of cancers compared to those over 30 in most common cancers studied, and in those over 40, in all common cancers except one—breast cancer in women—being the exception, where the age group 40–49 years had the best five-year net survival. However, the youngest patients in the 18–29-year-old age group had a better one-year net survival in breast cancer (Supplementary Figure S3) before displaying a worse five-year net survival compared with 30–39 and 40–49-year-olds. For breast cancer, this may reflect more aggressive tumour biology in patients under 30 in the longer term or a difference in detection of recurrences between age groups for breast cancer.

Our results highlight cancer types with poor survival in younger patients, namely brain/CNS, connective and soft tissue and lung cancers. In the 40–49-year age group, the poorest survival outcomes of the 10 most common cancers occurred in lung cancer in both men and women, with a five-year net survival of 17.2% in men and 18.1% in women. Interestingly, five-year net survival following a lung cancer diagnosis in males aged 18–29 was 85.9%, much better than those aged 30–49 years, but this was based on only 14 cases and should be interpreted with caution. Cancer Research UK has recognised brain and lung cancers as being two of several cancers of substantial unmet need due to limited improvements in survival and therapeutic options [24], highlighting the necessity for further research in these two areas.

It is important to note that survival is crucially dependent on stage at diagnosis, which we have not presented or discussed in detail within this review; further research would be helpful to better understand if any differential survival estimates in younger adults are explained by differing presentation at diagnosis. Aside from cervical cancer, no cancers are currently screened for in Northern Ireland in people under 50.

### 3.1. Treatment

For adult cancers, treatment protocols are the same regardless of patient age. Younger age has been associated with ‘overtreatment’ in colorectal cancer in all stages of disease [25,26], even when the evidence base for treatment is lacking and does not seem to result in improved survival. The tendency to overtreat younger patients may be influenced by patient request, clinician caution and the impression that younger patients can tolerate toxicity from treatment regimens. There is a lack of real-world data to inform treatment decisions in younger patients in cancers that have previously been considered diseases of older age groups. For example, conflicting cancer outcomes have been reported for early-onset colorectal cancer, with studies showing better, worse or similar survival in younger patients compared to older patients [27]. Multiple factors are likely contributing to a lack of clarity in this area, such as the inclusion or exclusion of inherited syndromes (for example Lynch syndrome, which is recognised to carry a favourable prognosis in early stage colorectal cancer [28]) An important area of research regarding early-onset cancers is the need to untangle the complex factors affecting survival such as inherited versus sporadic cases, stage of disease and pathology, as well as decision making by clinicians and patients and their influence on treatment uptake and survival for cancers that until now have been mostly studied in older age groups.

### 3.2. Early Diagnosis

Early diagnosis is key in improving outcomes from cancer. There is evidence that cervical cancer screening reduces mortality [29], representing a successful strategy for prevention and early detection in younger adult women. In the UK, only cervical cancer screening is offered to eligible adults aged under 50 as part of the national screening programme. There is currently no role for breast cancer screening in average-risk young women [30]. A modelling study in four European counties (Italy, The Netherlands, Finland and Slovenia) showed that adding breast cancer screening from the ages of 45 to 49 would result in more breast cancer deaths prevented and more life years gained but would also lead to an increased number of false-positive results [31]. A current trial is ongoing in the UK to evaluate an age extension to the NHS breast cancer screening programme to include women aged 47 to 49 [32].

The US Preventative Services Task force recommended reducing the screening age for colorectal cancer to 45 in 2021 [33], showing an awareness of the changing incidence profile for the disease. It has also been suggested that a shift to risk-based screening and risk stratification models should be considered for colorectal cancer [34,35], providing a possible indication of the direction of cancer screening in the future.

## 4. Survivorship

With breakthroughs in early detection and cancer treatment in the last few decades, survival rates for cancers are improving. This is leading to a growing population of cancer survivors who have complex physical, social and emotional consequences from a cancer diagnosis and treatment. A meta-review of qualitative research on adult cancer survivors (of all ages) was published in 2019, which included 60 reviews [36]. Breast and gynaecological cancers were strongly represented, accounting for nearly half of reviews within the study, but limited reviews exist for other common cancers such as colorectal, melanoma and haematological malignancies. Common themes in the review included areas highly relevant to young cancer survivors such as returning to work, psychological issues, sexuality, fertility and body image. A qualitative study undertaken in the USA and published in 2019 found that cancer patients diagnosed between the ages of 18 and 39 years identified key survivorship needs related to the ongoing physical, emotional and cognitive effects of treatment, navigating follow-up care, psychosocial concerns and adjusting to a new normal [37].

In the UK, children and young adults with cancer (up to the age of 24 years) have access to tailored services including psychological and social support and follow up/monitoring for late effects of treatment [38]. However, for adults aged over 24 years, cancer care is provided in adult centres where management is largely similar across all age groups, not taking into account differences in their specific care needs. Patients aged 25 to 39 years in the UK have reported a lack of guidance in navigating health and supportive care services and the need for more information regarding their diagnosis, side effects of treatment and lifestyle modification [39].

### Long-Term Side Effects of Treatment

Physical sequelae of cancer management include residual toxicities from treatment such as peripheral neuropathy [40] and altered bowel habit [41] as well as long-term complications including fatigue [42], sleep disorders [43], cardiotoxicity [44], endocrine disease including diabetes and thyroid dysfunction [45], diseases of renal function and bone metabolism [46] and secondary malignancy [47]. Recognition of cancer-related comorbidity in young patients is important as it may lead to poorer long-term medical and psychosocial outcomes [48]. Polypharmacy, or the use of multiple medications, has been observed in young adult cancer survivors [49].

Psychological sequelae of cancer treatment include reduced quality of life and mental health disorders. Quality of life among long-term cancer survivors (>5 years) compared to cancer-free population-based controls has been found to be worse in cancer survivors, particularly affecting those younger than 50 [50]. Cancer survivors within the adolescent and young adult (AYA) cancer population are at increased risk of mood disorders and anxiety disorders compared to cancer-free controls [51], and it is likely we can extrapolate this finding to survivors in their 30s and 40s. Premenopausal breast cancer survivors report worsening in body image and anxiety 3 years after treatment [52]. In addition, early-onset cancer survivors may also display adverse lifestyle behaviours, and a high prevalence of obesity and lack of adherence to dietary and exercise recommendations have been observed in breast and colorectal cancer survivors diagnosed before 50 years of age [53]. Addressing the physical consequences of cancer treatment alongside encouraging a healthy lifestyle is important for long-term survivorship care.

Given the potential decades of life left for these young patients, developing measures to improve their quality of life, such as physical activity interventions [54,55], management of sleep disorders [56], holistic needs assessment [57] and psychosocial care [58] would enable individual needs to be addressed and allow patients to access relevant support services. This in turn, could lead to an improvement of quality of life.

Evidence-based guidance for young adult cancer survivorship in early onset cancers previously considered diseases of older adults has not been developed. A symposium engaging multiple stakeholders was held in Canada in 2019 to develop recommendations for late effects screening and care in the young adult population [59]. The unique supportive care needs of this group of young patients are often not included in paediatric and adult care models currently, and more research is needed into optimising survivorship follow up and care for this cohort.

## 5. Fertility

Cancer treatments can reduce fertility or cause infertility, making this an important issue to consider in younger adults with cancer. Studies have reported variation in whether fertility counselling is offered to young patients newly diagnosed with cancer [60,61,62]. A 2019 Bowel Cancer UK survey of 1,295 young bowel cancer patients and caregivers found 41% of patients were dissatisfied with the information and support provided to them with regard to fertility and family planning [63].

Given the urgency of cancer treatment, there is a limited time window for discussion of fertility between patients and healthcare professionals, and information regarding fertility preservation may be difficult for patients to comprehend, especially alongside extensive information about their cancer, staging and treatment. Healthcare professionals have reported discussing these issues in detail but feel that patients often were overwhelmed with too much information, and understanding or recall of these conversations was limited [61].

Factors that have been identified as relevant to patients regarding decisions around fertility preservation are prioritising parenthood, focusing on cancer treatment, fear of future regret, survivorship quality of life, concerns for having children in the future, cultural/social issues and fear of disease progression [64]. Female cancer patients in particular have associated infertility with a loss of purpose and an impact on their identity [65,66]. There is variation between patients regarding whether they place a higher value on the risk of infertility or treating the cancer and survival as their main concern [64].

Fertility treatment has been demonstrated as a coping strategy. In a study of female breast cancer patients who had undergone fertility preservation, participants shared the difficulty of having a diagnosis of cancer along with a threat to fertility yet also felt the process of fertility treatment helped them cope with having breast cancer by focusing on their future and bringing about a strong survival instinct [67]. Patients have also described advocating for themselves in the area of fertility preservation. This included initiating conversations with healthcare professionals, facilitating communication between different departments and turning to the Internet for information [60].

Healthcare professionals have reported mixed awareness of fertility preservation treatments, with some lacking knowledge of what is involved, making conversations with patients more difficult [68]. Reported barriers for clinicians with regard to discussing fertility preservation are a lack of familiarity with the procedures and technologies, referral processes, perception of outcomes and a prioritisation for cancer treatment and survival [69]. There can also be differing perspectives between medical specialties in terms of who is responsible for discussing fertility preservation with patients [68]. The type and stage of cancer, along with a patient’s biological sex, are issues that are taken into account by healthcare professionals, as these factors will influence the timing and type of fertility preservation procedures available to patients [70].

Little is known regarding the impact of potential infertility concerns on the partners of cancer survivors and their relationships. A study investigating couple communication about fertility concerns after cancer found that open and honest communication resulted in feelings of support and relationship growth [71]. However, a minority of participants reported hiding their feelings in order to protect their partner, and this area was found to be a source of tension in some relationships. Cancer-related fertility concerns can place strain on relationships with feelings of stress, pressure and guilt manifesting in both patients and partners, in some cases contributing to relationship or marriage breakdown [72]. Other couples have reported struggling with cancer-related fertility concerns has strengthened their relationships [72].

## 6. Role of Social Media

The widespread use of social media in the 21st century has provided sources of information, advice, peer support and advocacy opportunities for cancer patients, particularly younger generations who have grown up with technology. In 2012, it was recognised that there was an evolving community of online cancer patients on Twitter exchanging information and engaged in psychological support [73]. A 2016 survey of 102 cancer patients aged 13–24 found that 41.6% rated the importance of digital communication to their lives as “essential” [74]. In 2017, a systematic review of social media and colorectal cancer recognised that cancer patients and their relatives are increasingly engaging with social media [75]. The COVID-19 pandemic has further served to normalise virtual activities and online communities, particularly among cancer patients who were asked to shield during the pandemic.

A survey of 45 young cancer patients aged 18–39 in 2020 found that many participants learned about social media support from a variety of sources, including recommendations from other individuals, Internet searches or advocacy organisations, and some describe an initial hesitance to get involved due to feeling overwhelmed [76]. There was also variety in the type of connections sought by patients, with some looking for information about symptoms, treatments and side effects, and others searching for primarily social purposes, building relationships with others in a similar position and providing or receiving emotional support.

Social media provides opportunities for patients and caregivers to share their own personal stories, fundraise for specific charities or causes, advocate for others in a similar position, raise awareness of cancer and break what are perceived as taboos in society such as embarrassing symptoms or end-of-life care. This has been reflected in the story of Dame Deborah James, a young colorectal cancer patient in the UK who chronicled her cancer journey using the handle @bowelbabe and had hundreds of thousands of followers on social media across different platforms.

Social media can be harnessed as a tool for campaigns, allowing messages to be carried around the world. Bowel Cancer UK is one of the leading bowel cancer charities in the United Kingdom and launched their “Never Too Young” campaign in 2013, which aims to give younger patients a voice and influence clinical practice and policy change to stop people dying of bowel cancer under the age of 50. This campaign has been adopted by other countries internationally and has a strong presence on social media with the hashtag #NeverTooYoung.

Social media has been described as a “paradigm shift” in the field of medicine, and the challenge for physicians and researchers is to utilise social media as a resource for research, as well as acknowledging the influence younger patients have in introducing new technology into healthcare [77]. However, studies have shown a minority of healthcare professionals engage with patients on social media, and scientific credibility of information on these platforms is a concern [78,79,80,81]. Online platforms carry the potential to be used in recruitment for studies, data collection, patient engagement, education and dissemination of results. The promise of social media in the early-onset cancer patient population has been demonstrated by qualitative studies [82], and Internet-based interventions for lifestyle modification in cancer survivors have also been piloted [83,84].

## 7. Future Research Directions

Our suggested important areas of future research in early-onset cancers are summarised in Table 1. Raising awareness of the observed trends regarding early-onset cancers is key in healthcare professionals, the scientific community and the general public. Encouraging individuals to be aware of potential cancer symptoms, seek medical assessment and attend cancer screening is vital to improve early detection. Enhancing knowledge of primary care physicians and removing barriers to referral such as age limits in cancer referral pathways will enable early investigation and referral for these patients. Advancing techniques in investigations allowing triage such as faecal immunohistochemical testing to select patients who proceed to colonoscopy is an example of a high impact, low-cost intervention for potential suspected cancer referrals, allowing efficient use of limited resources.

Detailed discussion about risk factors for each type of cancer are beyond the scope of this review. However, lifestyle factors such as alcohol, smoking and obesity which have been associated with several cancer types are likely to be contributing to some of the rise in early-onset cancers. Public health campaigns and encouragement of a healthy lifestyle could help to prevent development of cancer in some cases. Novel areas of research such as antibiotic usage, the gut microbiome and early life exposures in the perinatal period or childhood are also under investigation. While the majority of cases of early-onset cancers appear to be sporadic in nature, access to genetic testing in relevant cases is imperative, as germline mutations can still occur and have wider implications for cancer management and surveillance for the affected patient and their family members.

Effective treatment for early-onset cancers is important in improving cancer survival, particularly for cancers with poor outcomes such as lung, brain/CNS and connective and soft tissue cancers. Personalised medicine treatment strategies are needed for young patients to ensure the best possible outcomes, not just for cancer, but in terms of quality of life, long term toxicities of treatment and functional outcomes.

Specific care needs of these young adults should involve support around returning to work, mental health, communication with children around cancer and financial stress. In addition, end-of-life care needs of early-onset cancer patients are likely to include family support for young children who are about to lose a parent to cancer. Psychosocial support and input from a multidisciplinary healthcare team should address the unique holistic care needs of these younger patients, and the development of survivorship guidelines would standardise care across the UK.

## 8. Conclusions

Patients with early-onset cancers face unique challenges across the entire cancer process, from investigation and diagnosis, through treatment to survivorship or end-of-life care. Our review is narrative in nature and is not exhaustive, but we feel it highlights relevant areas for discussion in early-onset cancers. Prevention and early detection are key to reducing disease burden and improving cancer outcomes. As healthcare professionals and scientific researchers, we should be aiming to create treatment pathways for early-onset cancers where at each stage patients and their families are given autonomy and support relevant to their individual circumstances, where their concerns are addressed, and treatment is evidence-based and innovative. Finally, we should not underestimate the power of hope (indeed “rebellious hope”, according to the late Dame Deborah James) for young cancer patients and their families. We have the opportunity at each stage of the patient journey to contribute to more effective treatments, a better quality of life, living beyond cancer or meaningful palliative care.

## Figures and Tables

**Table 1:** Summary of future research areas for early-onset cancers.

Future Research Directions for Early-Onset Cancers
Raising awareness of observed trends among professionals and the public Removing barriers to referral Developing investigations allowing risk stratification/triage of young patients with cancer symptoms Increasing knowledge around potential risk factors Addressing modifiable risk factors Identifying individuals who would benefit from genetic testing Determining optimal treatment strategies to improve survival Investigating differences in incidence and survival according to stage at diagnosis Ensuring adequate information on fertility is provided to patients and fertility preservation treatment is offered to potentially eligible individuals Addressing specific supportive care needs (including but not limited to returning to work, mental health, communication with children around cancer and financial stress) across the whole patient journey: at diagnosis, during treatment and as part of survivorship care Optimising information provision and sharing online, including through social media

## Data Availability

Cancer statistics for Northern Ireland, on which this study was based, are available on the Northern Ireland Cancer Registry website: https://www.qub.ac.uk/research-centres/nicr/CancerInformation/official-statistics/ (accessed on 30 November 2021).

## Supplementary Materials

The following supporting information can be downloaded at: https://www.mdpi.com/article/10.3390/cancers14164021/s1, Table S1. Classification of cancer type using ICD10; Table S2: Cancer survival (ex NMSC) among 18 to 29 year olds: patients diagnosed in 2000–2014, followed up to the end of 2019; Table S3: Cancer survival (ex NMSC) among 30 to 39 year olds: patients diagnosed in 2000–2014, followed up to the end of 2019; Table S4: Cancer survival (ex NMSC) among 40 to 49 year olds: patients diagnosed in 2000–2014, followed up to the end of 2019; Figure S1: One-year and five-year net survival for all cancers (excluding non-melanoma skin cancer) for males and females by age group; Figure S2: One-year net survival for males by age group diagnosed in 2000–2014, followed up to end of 2019; Figure S3. One-year net survival for females by age group diagnosed in 2000–2014, followed up to end of 2019.

## Abbreviations

AYA	Adolescent and young adult
CI	Confidence interval
EAS	European age-standardised
CNS	Central nervous system
NICR	Northern Ireland Cancer Registry
NMSC	Non-melanoma skin cancer
