# Supplementary material for: Early-Onset Cancers in Adults: A Review of Epidemiology, Supportive Care Needs and Future Research Priorities
Source: Cancers (Basel). 2022 Aug 20;14(16):4021. doi: 10.3390/cancers14164021 (PMC9406462; doi:10.3390/cancers14164021)
Supplement: Supplementary file 1 [file cancers-14-04021-s001.zip › cancers-1825387-supplementary.pdf]

Table S1. Classification of cancer type using ICD10

**Cancer type**	**ICD10 code**	**Cancer type**	**ICD10 code**
Bladder	C67	Lung	C33-C34
Brain and CNS	C70-C72, C75.1-C75.3	Lymphoma	C81-C86
Cervical	C53	Melanoma	C43
Colorectal	C18-C20	Ovarian and fallopian tube	C56-C57.4
Connective and soft tissue	C47, C49	Prostate	C61
Breast	C50	Testicular	C62
Head and Neck	C00-C14, C30-C32	Thyroid	C73
Kidney	C64	Unknown primary	C77-C80
Leukaemia	C91-C95	Uterine	C54-C55

CNS: Central Nervous System

Table S2. Cancer survival (excluding non-melanoma skin cancer) among 18 to 29 year olds: patients diagnosed in 2000-2014, followed up to the end of 2019

Males					
Cancer type	**Number of patients included**	**One-year observed survival (95% CI)**	**Five-year observed survival (95% CI)**	**One-year net survival (95% CI)**	**Five-year net survival (95% CI)**
Testicular cancer	287	97.9% (95.4% - 99.1%)	95.1% (91.9% - 97.1%)	98.0% (96.3% - 99.7%)	95.5% (93.0% - 98.0%)
Lymphoma	115	97.4% (92.1% - 99.2%)	92.1% (85.3% - 95.8%)	97.5% (94.6% - 100.0%)	92.2% (87.4% - 97.3%)
Brain and central nervous system cancer	84	89.3% (80.4% - 94.3%)	65.3% (54.1% - 74.4%)	89.4% (83.0% - 96.2%)	65.6% (56.2% - 76.6%)
Melanoma	84	97.6% (90.8% - 99.4%)	94.0% (86.3% - 97.5%)	97.7% (94.5% - 100.0%)	94.3% (89.4% - 99.5%)
Leukaemia	45	88.9% (75.3% - 95.2%)	71.1% (55.5% - 82.1%)	89.0% (80.3% - 98.5%)	71.3% (59.4% - 85.7%)
Colorectal cancer	16	100%	81.3% (52.5% - 93.5%)	100%	81.7% (65.1% - 100.0%)
Head and neck cancer	23	100%	81.7% (58.2% - 92.7%)	100%	82.0% (67.6% - 99.6%)
Connective and soft tissue cancer	22	59.1% (36.1% - 76.2%)	40.9% (20.9% - 60.1%)	59.2% (42.2% - 82.9%)	41.0% (25.3% - 66.5%)
Thyroid cancer	11	100%	100%	100%	100%
Lung cancer	14	92.9% (59.1% - 99.0%)	85.7% (53.9% - 96.2%)	92.9% (80.7% - 100.0%)	85.9% (69.9% - 100.0%)
All cancers (ex NMSC)	778	93.3% (91.3% - 94.9%)	83.3% (80.5% - 85.8%)	93.4% (91.7% - 95.2%)	83.7% (81.1% - 86.4%)
Females					
Melanoma	191	99.5% (96.3% - 99.9%)	97.3% (93.7% - 98.9%)	99.5% (98.5% - 100.0%)	97.5% (95.2% - 99.8%)
Cervical cancer	205	96.1% (92.3% - 98.0%)	89.7% (84.6% - 93.1%)	96.1% (93.5% - 98.8%)	89.8% (85.8% - 94.1%)
Thyroid cancer	76	100%	100%	100%	100%
Lymphoma	101	94.1% (87.3% - 97.3%)	92.1% (84.8% - 96.0%)	94.1% (89.6% - 98.8%)	92.1% (87.0% - 97.5%)
Ovarian and fallopian tube cancer	93	95.7% (88.8% - 98.3%)	91.2% (83.2% - 95.5%)	95.7% (91.6% - 99.9%)	91.3% (85.7% - 97.3%)
Breast cancer	76	100%	78.8% (67.7% - 86.4%)	100%	78.9% (70.2% - 88.6%)
Brain and central	47	89.4% (76.3% - 95.4%)	64.8% (49.1% - 76.8%)	89.4% (81.1% - 98.5%)	64.9% (52.5% - 80.3%)

nervous system cancer					
Leukaemia	37	86.5% (70.5% - 94.1%)	72.7% (55.1% - 84.3%)	86.5% (76.3% - 98.1%)	72.7% (59.8% - 88.5%)
Colorectal cancer	18	94.4% (66.6% - 99.2%)	83.3% (56.8% - 94.3%)	94.5% (84.7% - 100.0%)	83.4% (68.2% - 100.0%)
Connective and soft tissue cancer	12	66.7% (33.7% - 86.0%)	50.0% (20.8% - 73.6%)	66.7% (45.6% - 97.5%)	50.1% (29.4% - 85.2%)
All cancers (ex NMSC)	929	94.8% (93.2% - 96.1%)	87.0% (84.6% - 89.0%)	94.9% (93.4% - 96.3%)	87.1% (85.0% - 89.3%)

Table S3. Cancer survival (excluding non-melanoma skin cancer) among 30 to 39 year olds: patients diagnosed in 2000-2014, followed up to the end of 2019

Cancer type	**Number of patients included**	**One-year observed survival (95% CI)**	**Five-year observed survival (95% CI)**	**One-year net survival (95% CI)**	**Five-year net survival (95% CI)**
Males					
Testicular cancer	314	99.4% (97.5% - 99.8%)	99.4% (97.5% - 99.8%)	99.5% (98.6% - 100.0%)	99.5% (98.6% - 100.0%)
Melanoma	143	97.9% (93.6% - 99.3%)	90.8% (84.7% - 94.6%)	98.0% (95.7% - 100.0%)	91.5% (86.9% - 96.4%)
Lymphoma	131	95.4% (90.1% - 97.9%)	88.5% (81.7% - 92.9%)	95.5% (92.0% - 99.2%)	88.9% (83.7% - 94.6%)
Colorectal cancer	96	82.1% (72.7% - 88.5%)	62.9% (52.3% - 71.7%)	82.2% (74.8% - 90.2%)	63.3% (54.3% - 73.9%)
Brain and central nervous system cancer	103	79.5% (70.4% - 86.2%)	49.1% (39.1% - 58.3%)	79.6% (72.2% - 87.8%)	49.5% (40.6% - 60.2%)
Leukaemia	56	94.6% (84.3% - 98.2%)	76.8% (63.4% - 85.8%)	94.8% (89.1% - 100.0%)	77.4% (67.1% - 89.2%)
Thyroid cancer	28	100%	96.4% (77.2% - 99.5%)	100%	96.8% (90.3% - 100.0%)
Kidney cancer	24	79.2% (57.0% - 90.8%)	70.8% (48.4% - 84.9%)	79.3% (64.9% - 96.9%)	71.4% (55.6% - 91.7%)
Head and neck cancer	41	87.8% (73.2% - 94.7%)	63.4% (46.8% - 76.1%)	87.9% (78.6% - 98.4%)	63.8% (50.7% - 80.2%)
Connective and soft tissue cancer	34	85.3% (68.2% - 93.6%)	76.5% (58.4% - 87.5%)	85.4% (74.4% - 98.0%)	77.0% (64.1% - 92.5%)
All cancers (ex NMSC)	1147	88.7% (86.8% - 90.4%)	77.0% (74.4% - 79.3%)	88.9% (87.0% - 90.7%)	77.5% (75.1% - 80.0%)
Females					
Breast cancer	782	97.6% (96.2% - 98.4%)	81.6% (78.7% - 84.1%)	97.6% (96.6% - 98.7%)	81.9% (79.2% - 84.7%)
Cervical cancer	439	97.5% (95.5% - 98.6%)	91.0% (87.9% - 93.4%)	97.6% (96.1% - 99.0%)	91.3% (88.7% - 94.1%)
Melanoma	310	99.7% (97.7% - 100.0%)	97.7% (95.3% - 98.9%)	99.7% (99.1% - 100.0%)	98.1% (96.4% - 99.7%)
Thyroid cancer	114	100%	99.1% (93.8% - 99.9%)	100%	99.4% (97.7% - 100.0%)
Ovarian and fallopian tube cancer	151	94.0% (88.9% - 96.9%)	84.7% (77.8% - 89.5%)	94.1% (90.4% - 97.9%)	84.9% (79.3% - 90.9%)
Colorectal cancer	80	93.8% (85.6% - 97.3%)	72.4% (61.2% - 80.9%)	93.8% (88.7% - 99.2%)	72.6% (63.5% - 83.1%)
Lymphoma	89	95.5% (88.5% - 98.3%)	92.1% (84.2% - 96.2%)	95.6% (91.4% - 99.9%)	92.5% (87.0% - 98.2%)

Brain and central nervous system cancer	52	80.8% (67.2% - 89.2%)	61.5% (47.0% - 73.2%)	80.8% (70.9% - 92.1%)	61.8% (50.0% - 76.4%)
Head and neck cancer	40	92.5% (78.5% - 97.5%)	90.0% (75.5% - 96.1%)	92.6% (84.8% - 100.0%)	90.1% (81.3% - 99.7%)
Lung cancer	40	62.5% (45.7% - 75.4%)	37.5% (22.9% - 52.1%)	62.5% (49.4% - 79.2%)	37.6% (25.5% - 55.6%)
All cancers (ex NMSC)	2353	94.3% (93.3% - 95.2%)	82.9% (81.3% - 84.4%)	94.4% (93.5% - 95.3%)	83.2% (81.7% - 84.8%)

Table S4. Cancer survival (excluding non-melanoma skin cancer) among 40 to 49 year olds: patients diagnosed in 2000-2014, followed up to the end of 2019

Cancer type	**Number of patients included**	**One-year observed survival (95% CI)**	**Five-year observed survival (95% CI)**	**One-year net survival (95% CI)**	**Five-year net survival (95% CI)**
Males					
Colorectal cancer	396	82.8% (78.7% - 86.2%)	55.3% (50.2% - 60.0%)	83.1% (79.4% - 86.9%)	56.2% (51.4% - 61.4%)
Melanoma	233	97.8% (94.9% - 99.1%)	87.9% (82.9% - 91.5%)	98.1% (96.2% - 100.0%)	89.2% (85.1% - 93.6%)
Head and neck cancer	245	84.5% (79.3% - 88.5%)	59.9% (53.4% - 65.7%)	84.7% (80.3% - 89.4%)	60.8% (54.9% - 67.4%)
Lymphoma	257	89.5% (85.1% - 92.7%)	83.2% (78.1% - 87.3%)	89.7% (86.1% - 93.6%)	84.5% (80.0% - 89.3%)
Kidney cancer	136	83.1% (75.7% - 88.4%)	70.6% (62.1% - 77.5%)	83.3% (77.2% - 89.8%)	71.6% (64.3% - 79.8%)
Prostate cancer	160	98.1% (94.3% - 99.4%)	86.1% (79.7% - 90.6%)	98.4% (96.3% - 100.0%)	87.6% (82.3% - 93.2%)
Lung cancer	224	39.7% (33.3% - 46.1%)	17.0% (12.4% - 22.2%)	39.9% (33.9% - 46.8%)	17.2% (12.9% - 23.0%)
Testicular cancer	215	96.7% (93.3% - 98.4%)	92.5% (88.1% - 95.4%)	96.9% (94.6% - 99.3%)	93.9% (90.4% - 97.5%)
Brain and central nervous system cancer	128	68.0% (59.1% - 75.3%)	30.2% (22.4% - 38.3%)	68.1% (60.5% - 76.7%)	30.6% (23.5% - 39.8%)
Leukaemia	101	90.1% (82.4% - 94.5%)	73.3% (63.5% - 80.8%)	90.3% (84.7% - 96.3%)	74.2% (66.0% - 83.4%)
All cancers (ex NMSC)	2760	78.2% (76.6% - 79.7%)	59.3% (57.5% - 61.1%)	78.4% (76.8% - 79.9%)	60.3% (58.4% - 62.2%)
Females					
Breast cancer	2769	98.2% (97.6% - 98.6%)	87.0% (85.7% - 88.2%)	98.3% (97.8% - 98.8%)	87.9% (86.6% - 89.2%)
Melanoma	380	99.2% (97.6% - 99.7%)	95.0% (92.3% - 96.8%)	99.4% (98.5% - 100.0%)	95.9% (93.7% - 98.1%)
Colorectal cancer	344	86.0% (81.9% - 89.3%)	61.9% (56.6% - 66.8%)	86.2% (82.6% - 89.9%)	62.6% (57.6% - 68.0%)
Ovarian and fallopian tube cancer	328	92.4% (88.9% - 94.8%)	72.5% (67.3% - 77.0%)	92.5% (89.7% - 95.4%)	73.2% (68.5% - 78.2%)
Cervical cancer	297	94.6% (91.4% - 96.7%)	80.4% (75.4% - 84.5%)	94.8% (92.2% - 97.4%)	81.1% (76.7% - 85.8%)
Uterine cancer	248	97.6% (94.7% - 98.9%)	89.1% (84.4% - 92.4%)	97.7% (95.9% - 99.7%)	90.0% (86.2% - 94.1%)
Thyroid cancer	120	100%	95.8% (90.3% - 98.2%)	100%	96.7% (93.2% - 100.0%)
Lung cancer	205	42.2% (35.3% - 48.8%)	17.9% (13.0% - 23.5%)	42.2% (36.0% - 49.6%)	18.1% (13.5% - 24.3%)

Lymphoma	197	92.9% (88.3% - 95.7%)	83.7% (77.7% - 88.2%)	93.0% (89.5% - 96.7%)	84.5% (79.5% - 89.9%)
Kidney cancer	91	80.2% (70.5% - 87.0%)	72.5% (62.1% - 80.5%)	80.3% (72.6% - 88.9%)	73.2% (64.6% - 83.0%)
All cancers (ex NMSC)	5602	91.0% (90.2% - 91.7%)	77.8% (76.6% - 78.8%)	91.1% (90.4% - 91.9%)	78.5% (77.5% - 79.7%)

Figure S1. One-year and five-year net survival for all cancers (excluding non-melanoma skin cancer) for males and females by age group

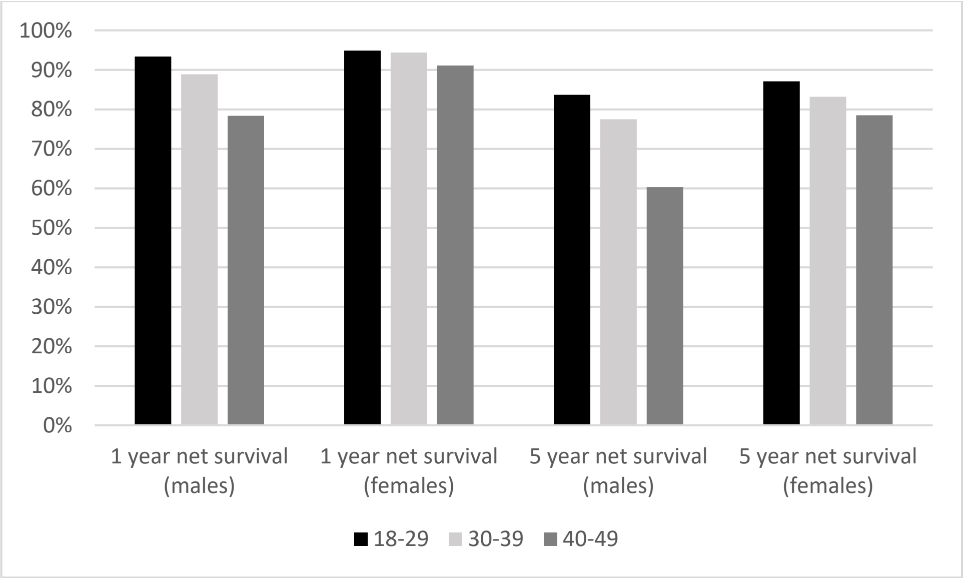

Figure S2. One-year net survival for males by age group diagnosed in 2000-2014, followed up to end of 2019*

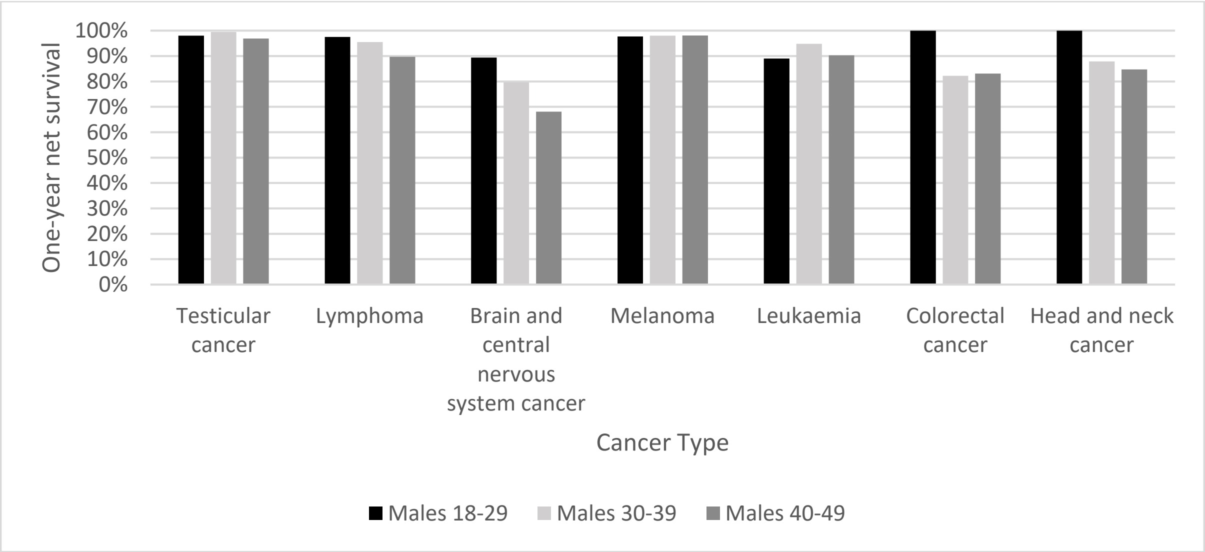

*Cancer types displayed are the cancers common to all three age groups in males

Figure S3. One-year net survival for females by age group diagnosed in 2000-2014, followed up to end of 2019*

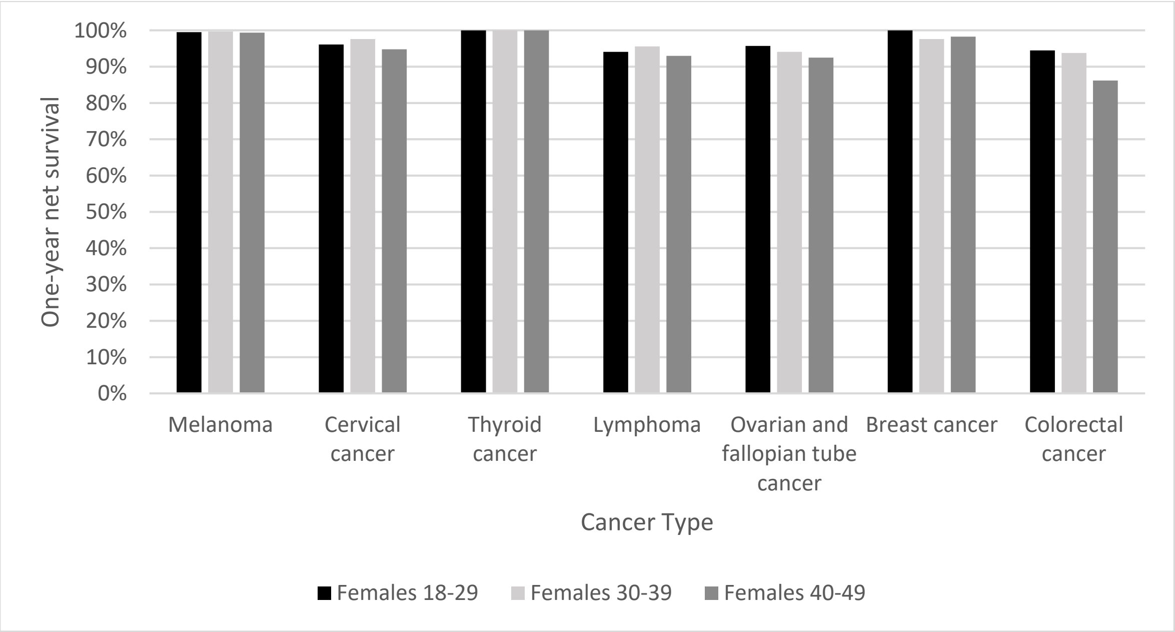

*Cancer types displayed are the cancers common to all three age groups in females
